# The Role of the Cerebellum in Tremor – Evidence from Neuroimaging

**DOI:** 10.5334/tohm.660

**Published:** 2021-11-15

**Authors:** Kevin R. E. van den Berg, Rick C. Helmich

**Affiliations:** 1 Centre of Expertise for Parkinson and Movement Disorders, Donders Institute for Brain, Cognition and Behaviour, Radboud University Medical Centre, Nijmegen, the Netherlands

**Keywords:** tremor, dystonia, Parkinson’s disease, cerebellum, magnetic resonance imaging, positron emission tomography

## Abstract

**Background:**

Neuroimaging research has played a key role in identifying which cerebral changes are associated with tremor. Here we will focus on the cerebellum, which may drive tremor oscillations, process tremor-related afferents, modulate activity in remote brain regions, or a combination.

**Methods:**

On the 6^th^ of October 2021, we conducted a PubMed search to select articles providing neuroimaging evidence for cerebellar involvement in essential tremor (ET), Parkinson’s disease (PD) tremor, and dystonic tremor (DT).

**Results:**

In ET, tremor-related activity is found in motor areas of the bilateral cerebellum, and altered functional connectivity within and outside the cerebellum correlates with tremor severity. Furthermore, ET is associated with cerebellar atrophy, but also with compensatory structural changes outside the cerebellum (e.g. supplementary motor area). In PD, tremor-related cerebellar activity and increased cerebello-thalamic coupling has been found. Emerging evidence suggests that the cerebellum plays a key role in dopamine-resistant rest tremor and in postural tremor. Cerebellar structural alterations have been identified in PD, but only some relate to tremor. DT is associated with more widespread cerebral networks than other tremor types.

**Discussion:**

In ET, the cerebellum likely acts as an oscillator, potentially due to loss of inhibitory mechanisms. In contrast, in PD the cerebellum may be a modulator, which contributes to tremor oscillations by influencing the thalamo-cortical system. The precise role of the cerebellum in DT remains unclear. We recommend that future research measures tremor-related activity directly by combining electrophysiology with neuroimaging, while brain stimulation techniques may be used to establish causality.

**Highlights:**

This review of neuroimaging studies has provided convincing evidence that the cerebellum plays a key role in the pathophysiology of ET, PD tremor, and dystonic tremor syndromes. This contribution may consist of driving tremor oscillations, processing tremor-related afferents, modulating activity in remote brain regions, or all the above.

## Introduction

Tremor is defined as an involuntary, rhythmic, oscillatory movement of a body part [1]. It is one of the most common movement disorders and can be an isolated symptom or part of a specific disease. Neuroimaging research has played a key role in understanding which brain areas are involved in tremor, and in defining how the interplay between these brain regions can result in tremor. Research has shown that multiple cerebral areas make up the oscillatory network which lays at the core of tremor generation. Structural and functional changes in the cerebellum are linked consistently to various types of tremor [2]. This is further supported by clinical studies showing that interventions in thalamic receiving nuclei of the cerebellum can suppress tremor [3]. One problem with neuroimaging studies is that they cannot show whether cerebellar activity is the cause or consequence of tremor. Furthermore, imaging techniques such as functional MRI (fMRI) lack the temporal resolution to detect changes at tremor frequency. Therefore, other research is needed to distinguish whether the cerebellum is the main tremor oscillator, whether it processes afferent input of the tremor itself or from other tremor-related brain regions, or a combination of these factors. Here, we aim to provide a review of neuroimaging evidence regarding the role of the cerebellum in the three most common clinical tremor syndromes: essential tremor (ET), tremor in Parkinson’s disease (PD), and dystonic tremor (DT).

## Methods

On the 6^th^ of October 2021, a PubMed search was conducted using the following terms: (“tremor/diagnostic imaging”[MeSH] OR “tremor/physiopathology”[MeSH] OR (“essential tremor/diagnostic imaging”[MeSH] OR “essential tremor/physiopathology”[MeSH] OR “essential tremor/pathology”[MeSH]) OR (“parkinson disease/diagnostic imaging”[MeSH] OR “parkinson disease/physiopathology”[MeSH] OR “parkinson disease/pathology”[MeSH]) OR (“dystonic disorders/diagnostic imaging”[MeSH] OR “dystonic disorders/physiopathology”[MeSH] OR “dystonic disorders/pathology”[MeSH])) AND “tremor*”[Title] AND “cerebell*”[Title/Abstract]. We filtered for articles written in English and performed on human subjects. Articles were included if they provided neuroimaging evidence for cerebellar involvement in ET, PD tremor, or DT. Neuroimaging was defined as MRI, positron emission tomography (PET), computed tomography (CT), magnetoencephalography (MEG), electroencephalography (EEG), and MR spectroscopy (MRS). Exclusion criteria were absence of neuroimaging in methodology, review articles, and case reports. This search yielded 485 articles, from which 118 articles were included based on title/abstract. A total of 58 articles were selected for this review. Specific hand-picked articles were included as they were recently published and did not yet include Mesh-terms or provided supportive information regarding the role of the cerebellum.

## Essential tremor

**Figure 1:**
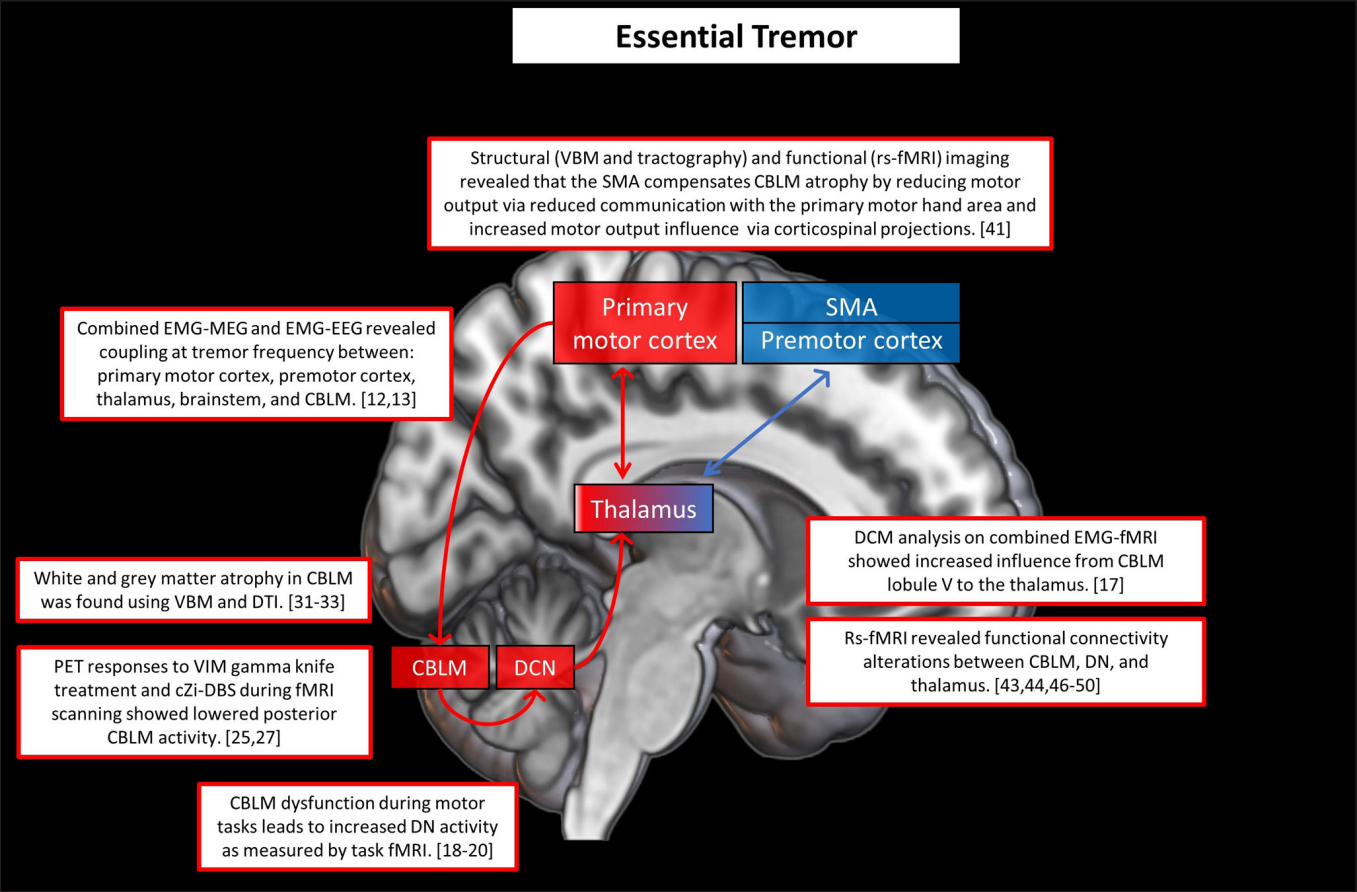
**Cerebral changes in essential tremor.** This figure illustrates a selection of key neuroimaging findings related to ET. The red areas are brain regions associated with (mainly) the cerebello-thalamo-cortical circuit and the blue areas are brain regions associated with (mainly) the basal ganglia circuit. The arrows depict the structural connections between these regions. The red and blue contours surrounding the boxes refer to the corresponding brain regions. Purple indicates involvement of both basal ganglia and cerebello-thalamo-cortical circuits. Number between brackets are references. Abbreviations: CBLM = cerebellum; DN = dentate nucleus; DCN = deep cerebellar nuclei; VIM = ventral intermediate nucleus; cZi = caudal zona incerta; SMA = supplementary motor area; DBS = deep brain stimulation; VBM = voxel-based morphometry; DTI = diffusion tensor imaging.

ET is defined as a bilateral action tremor of the arms, with or without an action tremor in other parts of the body [1]. The proposed definition of ET includes a 3-year history of tremor, excluding isolated head or voice tremors. A 3-year history was added to reduce the chance of development of other neurological symptoms such as Parkinsonism or ataxia [1]. If ET patients have additional rest tremor or neurological signs “of unknown significance”, then they are classified as “ET plus”. Under the umbrella of ET, different clinical subgroups have been identified, sometimes with underlying pathophysiological differences, such as ET with versus without resting tremor, late-onset ET, (or “aging-related tremor”) versus early-onset ET, and ET with versus without head tremor [4,5,6,7]. This shows that there are large clinical differences between ET patients, which are not always accounted for in existing neuroimaging studies. This may contribute to some of the variability in the findings discussed below. ***Figure 1*** provides an overview of the main neuroimaging findings in ET.

### Tremor-related activity in the cerebellum

The earliest suggestions regarding the role of the cerebellum in ET came from clinical observations where lesions in the thalamic ventral intermedius nucleus (VIM), a thalamic nucleus that receives input from the cerebellum, and cerebellar infarctions, abolished ET [8]. Prior studies involving harmaline-induced tremor in animals postulated that tremor was induced by rhythmic oscillatory activity originating from the inferior olivary nucleus (ION) in the brainstem. However, this hypothesis is much debated as many recent neuroimaging studies performed on human subjects have not consistently found abnormal activity in the ION. This has shifted the attention to the cerebellum, thalamus, and motor cortex [9]. Studies in the 1990s using PET and fMRI have provided the first neuroimaging evidence supporting the role of the cerebellum in tremor. These studies showed that unilateral involuntary postural tremor in ET patients was associated with bilateral cerebellar activation, as evidenced by increased cerebellar blood flow [8,10,11]. In contrast, mimicked tremor or passive wrist motion in ET patients and controls only induced ipsilateral cerebellar activation. These results suggest that ET involves bilateral cerebellar activation as opposed to unilateral activation. Subsequent studies have put more emphasis on the involvement of cerebral oscillations at tremor frequency. One of the first studies used electromyography (EMG) combined with MEG to test for cerebro-muscular and cerebro-cerebral coherence during postural tremor in ET [12]. MEG is an imaging technique which measures magnetic fields produced by the changing electrical gradients during the firing of neurons. This electrophysiological component is then used in statistical analyses to find coherent signal sources between brain regions (cerebro-cerebral coherence) or between brain regions and peripherally measured EMG (cerebro-muscular coherence). Coherence means that two (or more) signals have a consistent phase-relationship, which indicates that there is functional communication. Coupling at tremor-frequency revealed a network consisting of the contralateral primary motor cortex, premotor cortex, thalamus, brainstem, and ipsilateral cerebellum. A more recent study using combined EMG-EEG confirmed these observations and found a flow of oscillatory activity from the cerebellum directed to the sensorimotor cortex and EMG in ET and PD [13]. Using similar methods, the same group also showed differences in the cerebral oscillating network between two ET subgroups: early-onset versus late-onset ET. While tremor in early-onset patients was coherent with the well-known cortico-brainstem-cerebello-thalamo-cortical network, patients with late-onset ET showed a cortico-thalamic network only. This indicates a different role of the cerebellum between these two ET subgroups [5]. A comprehensive translational study in a mouse model of ET, together with high-density cerebellar EEG and post-mortem material in ET patients, has shown evidence for a model where deficient climbing fibers-to-Purkinje cell synapses, related to a primary insufficiency of glutamate receptor delta 2 (GluRδ2) proteins, may lead to excessive cerebellar oscillations that drive ET [14]. A recent subsequent study has shown that such excessive cerebellar oscillations are present in both familial and sporadic ET cases [15].

Another set of studies combined simultaneous EMG and fMRI to correlate spontaneous changes in tremor power to blood oxygen level dependent (BOLD) activity. Compared to PET and MEG, MRI achieves a higher spatial resolution, which helps to localize tremor activity in the cerebellum. Tremor-related activations were mainly found in bilateral somatomotor regions of the cerebellum, including left lobules V, VI, VIIb, and IX, as well as right lobules V, VI, VIIIa, and VIIIb [16]. In a subsequent combined EMG and fMRI study, dynamic causal modelling (DCM) was used to quantify effective connectivity, i.e. the influence of one region over the other [17]. This analysis showed that fluctuations in tremor amplitude were associated with an excitatory modulatory effect onto intrinsic connectivity in the cerebellum (lobule V) and the thalamus, as well as increased effective connectivity from the cerebellum (lobule V) onto the thalamus. This suggests that the cerebellum drives ET through the thalamus. Furthermore, reduced intrinsic cerebello-cortical functional connectivity was found (independent of the tremor), which was associated with increased tremor severity [17]. Similar findings were reported by a study that used a grip force task to evoke ET in the scanner. During this task, ET patients had reduced functional intrinsic connectivity between lobules I-V of the cerebellum and the primary motor cortex compared to PD patients and healthy controls [18]. Moreover, BOLD activity in the cerebellum did not correlate with 3-8 Hz oscillations, whereas activity in the primary motor cortex did. These two findings hint at cerebellar dysfunction in ET.

Indeed, task-specific fMRI studies have provided evidence that ET is associated with cerebellar deactivations when performing a motor task [18,19,20]. One of these studies showed that task-related dentate nucleus activation correlated positively with tremor severity, whereas a decrease in BOLD activity was found in widespread cerebellar as well as cortical regions during finger tapping [19]. More recently, widespread bilateral cerebellar deactivations in ET were found during posturing when compared to mimicked tremor in controls [20]. These findings suggest that dysfunction in bilateral cerebellar motor areas may lead to altered activity in the dentate nucleus, and – through its connections with the thalamus – to more widespread abnormal activity across the brain. Taken together, functional neuroimaging studies show evidence for increased cerebellar activity (and cerebello-thalamic connectivity) during tremor, but also for reduced cerebellar activity (and functional connectivity) during motor tasks. These findings suggest a complex pattern of cerebellar hyperfunction (driving the tremor) and dysfunction in ET.

### Intervention studies and neuroimaging

Intervention studies employing deep brain stimulation (DBS) or ethanol ingestion in ET have advanced our understanding of the underlying cerebellar mechanisms. One of the first neuroimaging studies involving ethanol ingestion compared ET patients with healthy controls during rest and right wrist extension [21]. Ethanol ingestion lowered bilateral cerebellar activity in both ET patients and healthy controls. Interestingly, ethanol mainly lowered contralateral cerebellar activity. Moreover, ethanol lowered tremor amplitude whereas frequency was unaffected. More recent work tested the effect of ethanol on ET using EMG and EEG, combined with MRI to locate coherent sources [22]. Coherence between cortical and muscle activity during involuntary tremor in ET was predominantly located over cerebral sensorimotor areas, whereas amplitude reduction following ethanol ingestion related with reduced cerebellar coherence, which peaked over the contralateral cerebellum. These studies suggest that the well-known beneficial effects of ethanol on ET may be mediated by the cerebellum. It is not entirely clear how ethanol exerts these effects, given its extensive effects on brain function. One possibility is that it stimulates GABAergic mechanisms, which are thought to be deficient in ET [23,24].

Stereotactic interventions in the outcome pathways of the cerebellum, including DBS and thalamotomy, have expanded our understanding of the functional interaction between the cerebellum and other cerebral regions. Gamma Knife treatment of the left VIM led to metabolic decreases in a network consisting of the left thalamus, left superior and middle temporal gyri, right cerebellum posterior lobe, left middle and inferior frontal gyri, and right middle and inferior frontal gyri [25]. Moreover, a recent VIM-DBS study showed that greater therapeutic tremor response was associated with stronger structural connectivity to the primary motor cortex and cerebellum [26]. These findings support the idea that the cerebellum is involved in a broader cortico-cerebellar network, which can be modulated at the level of the thalamus. Traditionally, the VIM is targeted in thalamotomy or DBS, but other sites such as the caudal zona incerta (cZi) have become more common in ET treatment. The cZi is a subthalamic region and it is suggested that pathological oscillations from the cerebellum may travel through the cZi to the thalamus and motor cortex [27]. A recent fMRI study involving cZi-DBS showed that involuntary postural tremor in ET was associated with a motor network involving the contralateral primary sensorimotor cortex, premotor cortices, SMA proper, thalamus and bilateral cerebellum (right lobules IV, V, VI, vermis, VIII and left VI) [27]. During posturing, cZi-DBS resulted in decreased activity in the primary sensorimotor cortex and ipsilateral cerebellar lobule VIII. These studies found that stereotactic interventions in cerebellar projection sites (thalamus and cZi) resulted in reduced posterior cerebellar activity, together with functional changes in other cortical areas. This shows that the role of the cerebellum in ET is more diverse than simply sending tremor oscillations down the cerebello-thalamo-cortical tract: if this were the case, then blocking these oscillations at the level of the thalamus or cZi would not alter oscillatory activity within the cerebellum. The fact that it does suggests that cerebellar activity in ET is also driven by (increased) tremor-related sensory input, altered cortico-cerebellar projections, or a combination.

### Structural alterations in the cerebellum

Post-mortem pathological studies have provided evidence for degenerative changes in the cerebellum, such as a reduced number of Purkinje cells as well as axonal changes such as swellings or “torpedoes” [28,29,30]. MRI techniques such as diffusion tensor imaging (DTI) and voxel-based morphometry (VBM) allow the investigation of in-vivo structural alterations in ET. Various VBM and DTI studies have found white and grey matter atrophy in both cerebellar hemispheres [31,32,33]. Of note, one VBM study compared ET patients with both head and arm tremor to those with only head tremor and found significant atrophy in the bilateral cerebellum and vermis only in ET patients with head tremor [34,35].

These findings, amongst other results, suggest that different ET phenotypes are associated with different patterns of cerebral abnormalities. In the most recent consensus statement, ET patients with additional symptoms are referred to as “ET plus”, but this term is much-debated, and many people see it as a temporary label en-route to a more definitive diagnosis [1,36,37]. In line with this viewpoint, one study showed that ET patients exhibiting ataxia had smaller volumes of vermis lobule VI compared to ET patients without ataxia [38].

Specific areas of cerebellar degeneration may be related with specific disease features in ET. Familial ET patients exhibited degenerative changes in the dentate nucleus and superior cerebellar peduncle when compared to PD patients and healthy controls [39]. Degeneration of the dentate nucleus was able to completely distinguish familial ET from PD patients and healthy controls. In addition, familial ET with longer disease durations showed more degeneration in the dentate nucleus when compared to shorter durations. As there were no differences between dentate nucleus degeneration in PD patients and healthy controls, one could argue that dentate nucleus degeneration is a specific feature found in familial ET. Another study found more degenerative changes in the superior cerebellar peduncle in late-onset ET as compared to early-onset ET, which may be related to the faster rate of tremor progression in late-onset ET [40].

Degenerative cerebellar changes may also lead to compensatory alterations outside the cerebellum. One study found that ET patients had bilateral atrophy in lobules IV/V and VIII as well as increased grey matter volume in the bilateral SMA [41]. Grey matter volume in the SMA proper correlated negatively with grey matter atrophy in the contralateral cerebellum. In addition, the SMA had lower amplitudes of spontaneous neuronal fluctuation, less connectivity to the primary motor hand area, and a higher probability of connection to the spinal cord. Of note, structural and functional alterations in the SMA, as opposed to the cerebellum, correlated with clinical severity. These findings suggest that cerebellar atrophy in ET is compensated by the SMA proper, which attempts to reduce motor output by reduced communication with the primary motor hand area, as well as increased influence on motor output via corticospinal projections. This might also be true for tremor frequency, as grey matter volume in cerebellar vermis VIII correlated positively with tremor frequency, whereas grey matter volume in the SMA proper correlated negatively with tremor frequency. Additional compensatory mechanisms may also exist within the cerebellum. One study in ET patients aged 65 years or younger found bilateral cerebellar expansion, which may explain the slower disease progression in early-onset ET as cerebellar compensatory mechanisms are more effective in this group [42]. Taken together, there is much evidence for primary cerebellar pathology in ET, which is accompanied by structural alterations and compensation outside the cerebellum.

### Resting state alterations in the cerebellum

Resting-state (rs) fMRI is a widely used imaging technique that allows investigation of large-scale functional networks by means of temporal correlation of spontaneous low frequency fluctuations of BOLD signal, in the absence of a task. Individuals are usually asked to relax and stay awake with their eyes open. This means that there is no (action) tremor present during scanning. While this makes it easier to collect data, it is also more difficult to relate rs-fMRI changes in ET directly to the tremor itself: like structural MRI, any group difference may represent a “trait” that co-occurs with tremor but is not involved in tremor pathophysiology.

Rs-fMRI studies have found reduced functional connectivity in both anterior and posterior parts of the cerebellum [43]. As part of a cerebellar network, these areas also showed decreased connectivity with the sensorimotor and posterior default-mode network [43]. Decreased connectivity in the cerebellum correlated negatively with tremor scores [43,44]. Moreover, cognitive scores correlated negatively with connectivity alterations in the cerebellar network [44]. In addition to cognitive scores, clinical depression scores in depressed ET patients also correlated with posterior cerebellum (lobules IX) connectivity changes [45]. These findings put the cerebellum in a broader perspective, where it may play a role in both motor and non-motor symptoms in ET.

The somatomotor parts of the cerebellum project, via the VIM, to the primary motor cortex, and functional connectivity between cerebellum and VIM has extensively been studied. One study showed that VIM-related functional connectivity was decreased in the bilateral cerebellum and tremor scores correlated negatively with bilateral cerebellar connectivity values [46]. However, other studies have provided evidence for an increased functional connectivity between the bilateral cerebellar lobules with the bilateral thalami which correlated positively with higher tremor scores [47,48]. Of note, disease duration also correlated positively with thalamo-cerebellar functional connectivity, which suggests that abnormal cerebello-thalamic connectivity is inherent to ET. The role of cerebello-thalamic connectivity in ET is further demonstrated by an intervention study using stereotactic radiosurgical thalamotomy [49]. This intervention reduced connectivity between the thalamus and motor cerebellum (lobule V), which correlated with lower global tremor scores (ADL-score). This further denotes the involvement of cerebello-thalamic connectivity in tremor severity.

Finally, connectivity changes in the dentate nucleus were also found [50]. Compared to healthy controls, functional connectivity between the dentate nucleus and the cerebellar cortex as well as with the thalamus was reduced in ET. Interestingly, dentate nucleus functional connectivity with the cerebellar cortex correlated positively with tremor amplitude, whereas dentate nucleus functional connectivity with the thalamus correlated negatively with tremor amplitude and disease duration. Moreover, dentate nucleus functional connectivity with the thalamus correlated positively with MoCA scores. This implies that connectivity alterations between the dentate nucleus, cerebellar cortex, and thalamus are inherent to ET pathophysiology and relate to cognitive and motor symptoms.

### Evidence for GABAergic dysfunction

Evidence for GABAergic dysfunction comes from the clinical observation that several tremor-alleviating drugs stimulate the GABAergic system. In addition, action tremor could be induced in mice in which the gene encoding for the GABA_A_-receptor was knocked out [51]. PET-studies involving 11C-flumazenil found increased binding in the VIM, right dentate nucleus, cerebellar vermis, bilateral posterior lobes, and right anterior lobe in ET as compared to controls [23,52]. In addition, cerebellar 11C-flumazenil uptake correlated positively with tremor scores [52]. This may support the hypothesis that ET patients have decreased cerebellar GABA concentrations. However, increased 11C-flumazenil binding could also be a consequence of GABA_A_-receptor upregulation, which may be triggered by loss of GABA-containing Purkinje cells. One in vivo MRS study quantified GABA concentrations in the dentate nucleus and failed to show differences of GABA concentrations between ET and controls [53]. Another reason for the null-finding could be related to terminal sprouting, which occurs when remaining damaged Purkinje cells try to compensate their synaptic loss by compensatory sprouting of their terminal boutons [53]. Taken together, these findings suggests that GABAergic dysfunction may pay a role in the pathophysiology of ET.

## Tremor in Parkinson’s disease

**Figure 2:**
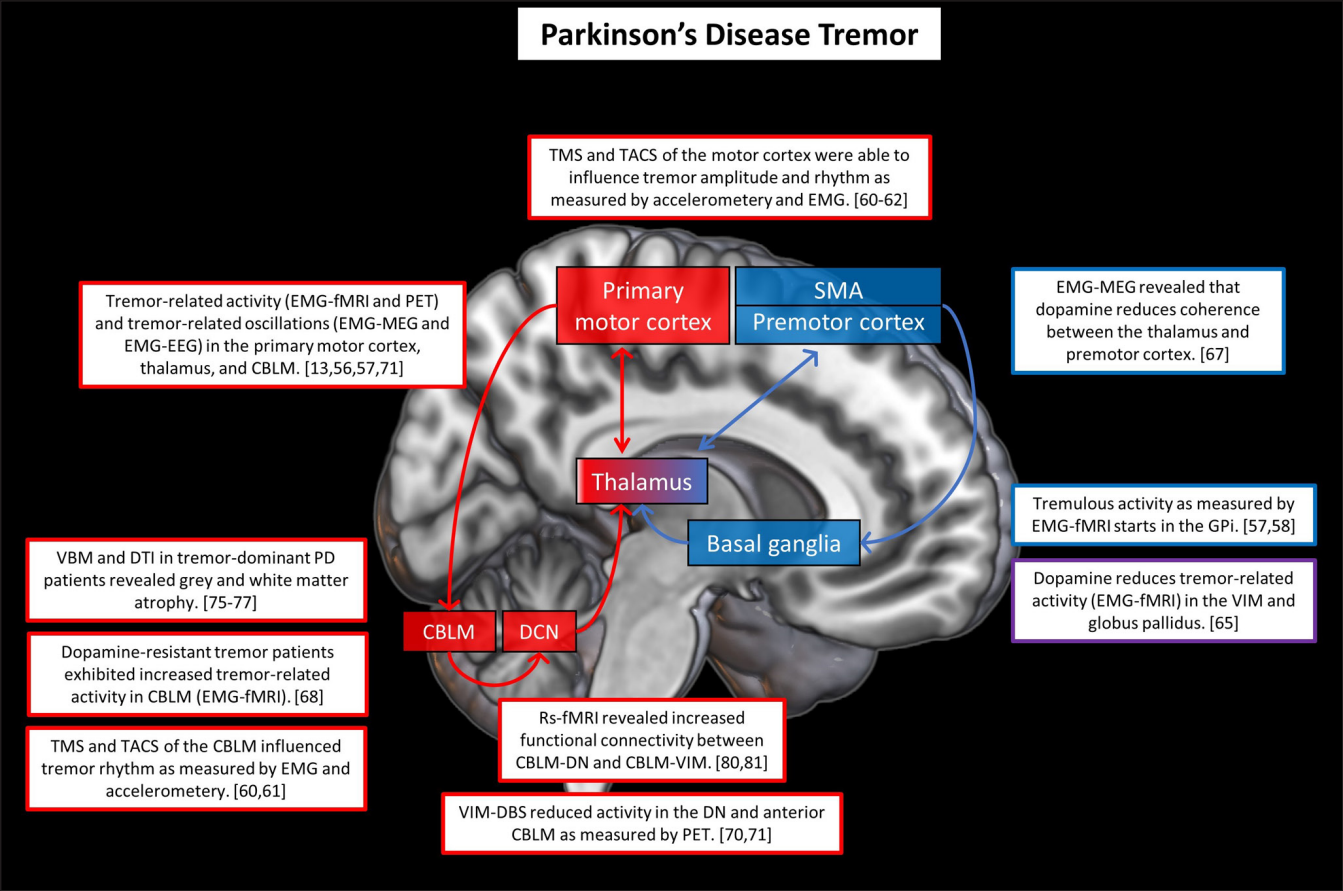
**Cerebral changes in Parkinson’s disease tremor.** This figure illustrates a selection of key neuroimaging findings related to PD tremor. The red areas are brain regions associated with (mainly) the cerebello-thalamo-cortical circuit and the blue areas are brain regions associated with (mainly) the basal ganglia circuit. The arrows depict the structural connections between these regions. The red and blue contours surrounding the boxes refer to the corresponding brain regions. Purple indicates involvement of both basal ganglia and cerebello-thalamo-cortical circuits. Number between brackets are references. Abbreviations: CBLM = cerebellum; DN = dentate nucleus; DCN = deep cerebellar nuclei; GPi = internal globus pallidus; VIM = ventral intermediate nucleus; cZi = caudal zona incerta; TACS = transcranial alternating current stimulation; TMS = transcranial magnetic stimulation; DBS = deep brain stimulation; VBM = voxel-based morphometry; DTI = diffusion tensor imaging.

PD harbours several types of tremor [54]. The classical PD tremor is a 4–6 Hz rest tremor, which is (transiently) reduced by a brisk voluntary movement of the tremulous limb. In many cases, the tremor re-emerges during stable posturing, at the same frequency [55]. In addition, a minority of patients have a pure postural tremor, or a kinetic tremor, at a higher frequency (±8–10 Hz) [54]. Most neuroimaging research has been done in rest tremor. ***Figure 2*** provides an overview of the main neuroimaging findings in PD tremor.

### Tremor-related activity in the cerebellum

**Figure 3:**
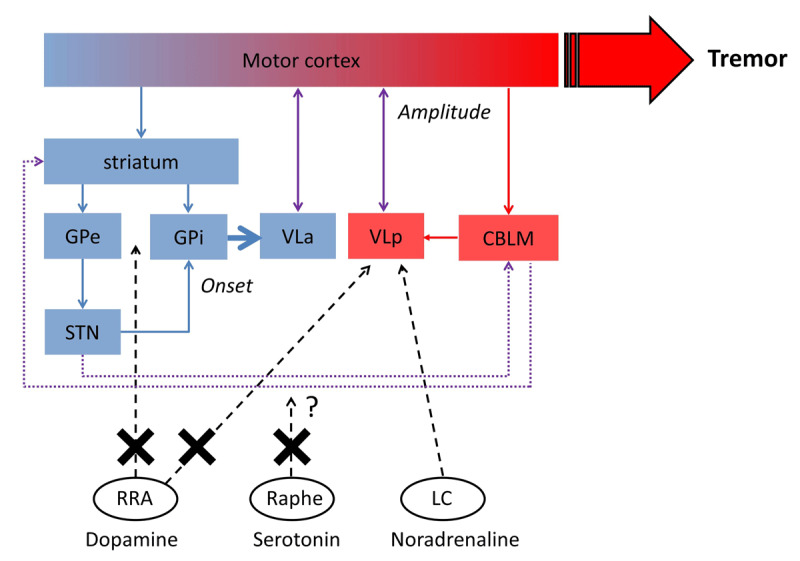
**The dimmer-switch model of Parkinson’s disease tremor.** Tremor related activity has been found in both the basal ganglia (depicted in blue) and the cerebello-thalamo-cortical circuit (depicted in red). Both circuits converge in the motor cortex (depicted in purple). Blue and red arrows indicate connections within each circuit; purple arrows indicate connections between the basal ganglia and cerebello-thalamo-cortical circuits. The open circles indicate neurotransmitter systems that project to these circuits and where changes have been reported in tremor-dominant PD. These include reduced dopaminergic projections from the RRA, reduced serotonergic projections from the raphe nuclei, and increased noradrenergic projections from the LC. In italics, hypothesized roles of nodes of this network in generating tremor that trigger the onset of tremor (GPi), analogous to a light switch, and maintain tremor amplitude (the cerebello-thalamo-cortical circuit), analogous to a light dimmer. VLa = ventrolateral anterior thalamus; VLp = ventrolateral posterior thalamus; CBLM = cerebellum; RRA = retrorubral area; LC = locus coeruleus. Modified with permission from Rick Helmich (Helmich RC. The cerebral basis of Parkinsonian tremor: a network perspective. Movement Disorders. 2018 Feb;33(2):219–31).

Tremor-related activity in PD rest tremor has primarily been found in the ipsilateral cerebellum (relative to the most affected hand), contralateral sensorimotor cortex, and contralateral thalamus (VIM) [56,57,58]. Early work involving simultaneous MEG and peripheral EMG measurements showed strong coherence between tremulous EMG activity and the contralateral primary motor cortex (M1) [56]. Furthermore, the M1 showed significant coupling at double tremor frequency with the SMA, posterior parietal cortex, somatosensory cortex, thalamic region, and cerebellum. The fact that there was only sparse direct coupling between the cerebellum and EMG signals, in contrast to strong M1-EMG coupling, suggests that not all cerebellar activity is explained by (tremor-related) somatosensory afferents [56]. Subsequent studies employed combined EMG-fMRI to study tremor-related activity [57,58]. As fMRI does not have the temporal resolution to measure cycle-by-cycle changes, spontaneous fluctuations in tremor amplitude (over multiple seconds) were correlated to cerebral activity. These studies also found tremor-related activity in the ipsilateral cerebellum (lobules V and VI), motor cortex (Brodmann Areas 4 and 6), and the thalamus (VIM) [57,58]. Tremor-related activity in both M1 and cerebellum correlated with clinical tremor scores, suggesting that both regions have a role in tremor amplitude [57]. The finding that spontaneous increases in tremor power during scanning were associated with brain activity in the globus pallidus, and the finding of increased coupling between the internal globus pallidus (GPi) and the M1 in tremor-dominant versus non-tremor PD patients, suggested that the basal ganglia have a role in triggering tremor. This gave rise to the “dimmer-switch hypothesis”: while the basal ganglia may trigger tremor (analogous to a light switch), the cerebello-thalamo-cortical circuit is thought to amplify tremor amplitude (analogous to a light dimmer; see ***Figure 3***). Further evidence for this idea came from dynamic causal modelling of EMG-fMRI data, which showed that spontaneous tremulous activity first emerges in the globus pallidus and is then relayed to the cerebello-thalamo-cortical-circuit through M1 [58].

While the findings above have shown that brain activity in the cerebello-thalamo-cortical circuit co-fluctuates with tremor power, these data cannot distinguish whether the activity is a cause or consequence of tremulous activity. Within the cerebello-thalamo-cortical circuit, cerebellar activity may not be involved in driving tremor amplitude, but instead it may reflect processing of sensory afferents from the tremulous limbs, or modulating of activity in e.g. the thalamus. This is supported by studies showing that PD tremor can occur in a patient with a resected cerebellum (albeit at a different frequency and a different phenotype), that cerebellar transcranial magnetic stimulation (TMS) resets PD re-emergent tremor but not resting tremor, and that cerebellar transcranial alternating current stimulation (TACS) changes the phase but not the amplitude of PD resting tremor [59,60,61]. This does not mean that cerebellar activity is trivial: somatosensory afferences may have a role in stabilizing the tremor rhythm, and these effects may be mediated either by the cerebellum (through climbing fibres and mossy fibres), by the thalamus (tremor cells in the VIM often also receive proprioceptive input), or both. It is unlikely that the role of the cerebellum in tremor consists *only* of afferent processing: in a combined EMG-MEG study, there was only sparse direct coupling between the cerebellum and EMG signals, in contrast to strong M1-EMG coupling [56]. In contrast to the cerebellum, the VIM and M1 probably have a more direct role in modulating tremor amplitude: there is evidence that TACS and TMS over M1 both reduce tremor amplitude, and stereotactic interventions in the VIM have strong anti-tremor effects [60,62,63].

### Effects of dopamine on PD resting tremor

Clinically, PD resting tremor has a variable response to dopaminergic medication. In 30 of 76 tremor-dominant PD patients (39%), resting tremor did not respond at all to a levodopa challenge, while the remaining 46 patients (61%) showed an excellent response (64–81% reduction in tremor amplitude) [64]. A combined EMG-fMRI study showed that dopaminergic medication reduced tremor-related activity in the VIM and globus pallidus, but not in the cerebellum or M1 [65]. This fits with findings that there are dopamine receptors in the thalamus and globus pallidus, but not in the cerebellum [66]. It also fits with another EMG-MEG study showing that dopaminergic medication specifically reduced the coherence strength between thalamus and premotor cortex, together with tremor power [67]. This suggests that thalamic dopamine depletion may contribute to the emergence of PD resting tremor, but this does not explain dopamine-resistant tremor. A comparison of dopamine-resistant and dopamine-responsive PD tremor showed that patients with dopamine-resistant tremor had more tremor-related activity in the cerebellar lobules IV, V, vermis IX, and in deep cerebellar nuclei (nucleus fastigii and interposed nucleus) [68]. In addition, patients with dopamine-resistant tremor showed less tremor-related activity in the VLpv and the somatosensory area OP4, as well as reduced functional connectivity between VLpv and OP4. Taken together, the involvement of the cerebellum in PD resting tremor is well established and its role may be more evident in dopamine-resistant tremor. Dopamine-resistant PD tremor may be explained by increased cerebellar and reduced somatosensory influences on the cerebellar receiving nucleus of the thalamus, making it less susceptible to the effects of dopamine. Nuclear imaging studies may further test how dopamine depletion outside the striatum (e.g. pallidum and thalamus) contributes to PD tremor. While a first DAT-SPECT study showed pallidal dopamine depletion in tremor-dominant PD patients versus non-tremor patients, a (larger) subsequent study could not replicate this [57,69].

Functional neuroimaging has provided additional insights in the areas specifically involved in PD tremor amplitude versus tremor frequency. VIM stimulation has been found to reduce activity in the contralateral (relative to stimulation site) dentate nucleus and the anterior lobe of the cerebellum [70,71]. Interestingly, activity in this cerebellar region did not correlate with tremor amplitude. Instead, activity in the ipsilateral sensorimotor cortex and anterior SMA did correlate positively with tremor amplitude. In line with these observations, studies employing TACS and TMS in PD rest tremor found that cerebellar stimulation did not influence tremor amplitude whereas M1 stimulation did [60,61,62]. Conversely, the cerebellum may play a cardinal role in modulating tremor frequency. VIM stimulation resulted in reduced contralateral dentate nucleus activity, which correlated negatively with tremor frequency [70]. In addition, both cerebellar TACS and TMS were able to modulate tremor rhythm [60,61]. This suggests that the individual nodes within the cerebello-thalamo-cortical-circuit have specific roles in relation to PD tremor amplitude and frequency.

### Structural alterations in the cerebellum

Structural imaging studies have investigated differences between two PD phenotypes: tremor-dominant versus akinetic-rigid phenotype. Clinically, the distinction between these subtypes is not set in stone, and different subtyping schemes have been proposed [72,73]. Also, it is relevant to note that the presence/absence of tremor is by no means the only difference between these subtypes: cognitive and non-motor deficits tend to occur more in non-tremor PD patients [74]. Hence, structural brain differences between subtypes may be related to other clinical features than tremor. Compared to the akinetic-rigid phenotype, tremor-dominant patients have reduced grey matter in cerebellar areas that are related to hand and arm movements, such as lobules IV, VI and VIIb, as well as the cerebellar vermis [75,76]. Furthermore, tremor-dominant patients had increased mean diffusivity along white matter tracts in the superior, middle, and inferior cerebellar peduncles, as well as the thalamus, which reflects altered microstructural integrity within the cerebello-thalamo-cortical-circuit [77]. In addition, cerebellar atrophy patterns and white matter alterations differed between PD patients and ET patients [78,79]. More specifically, ET exhibited reduced white matter integrity in the three cerebellar peduncles when compared to PD [78]. Furthermore, ET patients had reduced volumes of deep cerebellar nuclei whereas PD patients had smaller volumes in lobule VI [79]. Cerebellar volume has also been found to correlate to tremor severity. Postural tremor severity in ET increased with lower volumes in lobule VIII, whereas PD resting tremor severity increased with increasing volumes in lobule IV [79]. In closing, cerebellar atrophy in motor areas and microstructural changes in white matter tracts connecting the cerebellum with the rest of the brain have been found in PD, and some of these changes relate to PD resting tremor.

### Resting state alterations in the cerebellum

Resting state alterations have consistently been found in the cerebellum as well as its associated nuclei. Functional connectivity between the bilateral dentate nucleus and bilateral cerebellar anterior and posterior lobes was found to be increased in tremor-dominant PD patients. Moreover, tremor scores correlated positively with dentate nucleus-posterior cerebellar lobe connectivity. The opposite was true for functional connectivity between the dentate nucleus and prefrontal cortex, which was negatively correlated with tremor scores [80]. It was proposed that tremor may be related to decoupling of the prefrontal cortex with the dentate nucleus and increased coupling of the posterior cerebellum with the dentate nucleus. Similarly, tremor-dominant PD is associated with higher functional connectivity of the VIM with the cerebellum and M1 [81]. Again, functional connectivity of the VIM with the cerebellum and M1 correlated positively with tremor scores. In contrast, enhanced functional connectivity between the VIM and cerebellum was not found in the akinetic-rigid phenotype. This suggest that increased coupling of the cerebellum with the VIM and dentate nucleus plays a cardinal role in PD tremor pathogenesis. Additional findings from rs-fMRI consistently show altered spontaneous neuronal activity in the cerebellum, which correlates positively with tremor scores [82,83]. The fact that thalamotomy reduces spontaneous neuronal synchronization in the contralateral cerebellum further denotes its involvement in PD tremor [84].

## Dystonic tremor

**Figure 4:**
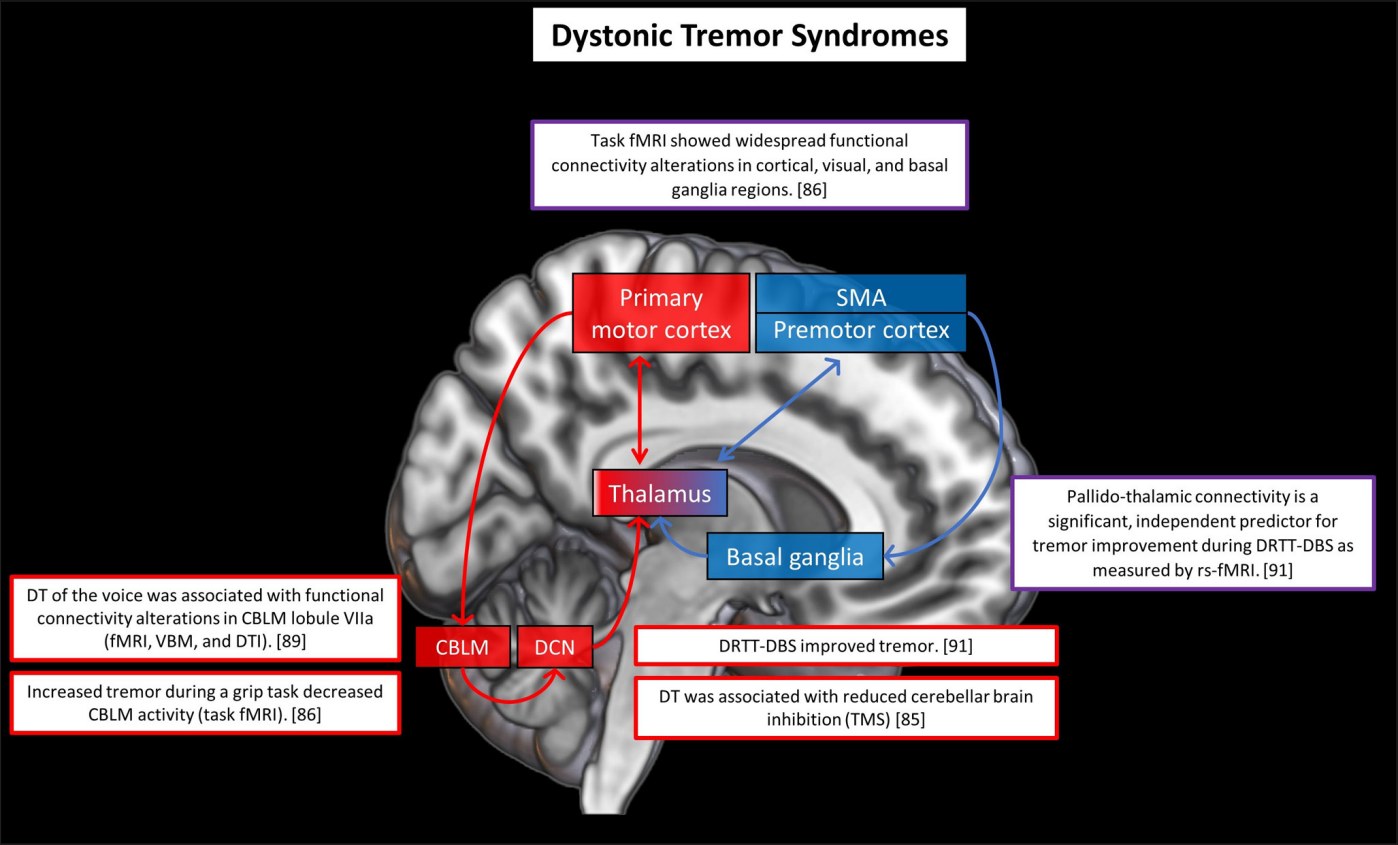
**Cerebral changes in dystonic tremor syndrome.** This figure illustrates a selection of key neuroimaging findings related to dystonic tremor syndrome (DTS). The red areas are brain regions associated with (mainly) the cerebello-thalamo-cortical circuit and the blue areas are brain regions associated with (mainly) the basal ganglia circuit. The arrows depict the structural connections between these regions. The red and blue contours surrounding the boxes refer to the corresponding brain regions. Purple indicates involvement of both basal ganglia and cerebello-thalamo-cortical circuits. Number between brackets are references. Abbreviations: CBLM = cerebellum; DCN = deep cerebellar nuclei; DBS = deep brain stimulation; DRTT = dentato-rubro-thalamic tract; VBM = voxel-based morphometry; DTI = diffusion tensor imaging; TMS = transcranial magnetic stimulation.

DT is defined as tremor in a body part affected by dystonia [1]. Examples include head tremor in cervical dystonia or segmental tremulous dystonia in the upper limbs. When tremor and dystonia occur simultaneously in different body parts, it is defined as tremor associated with dystonia [1]. Electrophysiological studies have shown differences between DT and tremor associated with dystonia. For example, DT patients had a more abnormal reduction in cerebellar inhibition compared to controls and ET patients, while this difference was not found for patients with tremor associated with dystonia [85]. It can be difficult to distinguish ET from DT. Especially cervical dystonia patients often have a tremor that resembles ET, i.e. a regular and symmetric action tremor of both arms, sometimes combined with head tremor [86]. It has been hypothesized that the cerebellum plays a cardinal role in DT, but there is little evidence [87]. ***Figure 4*** provides an overview of the main neuroimaging findings in DT.

### Dysfunctional activity and connectivity

Cerebellar involvement in DT may primarily involve widespread functional changes, as extensive structural alterations of the cerebellum have not been identified. In contrast to ET, structural alteration in DT patients have mainly been found outside the cerebellum [88,89]. Functional alterations in the sensorimotor network have been identified in patients with spasmodic dysphonia, a type of laryngeal dystonia, with or without additional DT of the voice [89,90]. Interestingly, patients with both spasmodic dysphonia and DT of the voice showed additional functional alterations in the cerebellum (VIIa). This suggests that dystonia and DT may be part of the same pathophysiological spectrum. This might also be true for ET and DT, based on similar symptomology and cerebellar involvement [89].

Even though ET and DT may appear similar on a symptom-level, connectivity measures in DT are more extensive compared to ET. In a grip force task, both ET and DT patient cohorts showed increased tremor with increased visual feedback [86]. Associated BOLD amplitudes in the cerebellum were reduced in both patient groups [86]. However, DT was characterised by more extensive functional connectivity reductions and changes in multiple cerebellar clusters, whereas ET only exhibited minor cortical functional connectivity reductions [86]. DT also involved widespread functional connectivity reductions in cortical, visual, and basal ganglia regions, which suggests involvement of distinct network-level connectivity in the cerebello-basal ganglia-cortical network [86]. A recent DBS study involving DT and ET also provided evidence for cerebellar involvement in DT pathophysiology [91]. DBS of the dentato-rubro-thalamic tract (DRTT), connecting the dentate nucleus of the cerebellum with the VIM, correlated with tremor improvement in both DT and ET. However, the connectivity pattern of the DRTT alone was not a significant predictor for tremor improvement. DBS of the pallido-thalamic tracts, connecting the basal ganglia with the thalamic ventralis oralis posterior (VOp) nucleus, correlated with tremor improvement in DT. In addition, pallido-thalamic connectivity was a significant, independent predictor for tremor improvement in DT. Effective stimulation in DT was associated with greater functional connectivity in the SMA, premotor cortex and associative prefrontal regions whereas greater functional connectivity to the primary motor cortex was found in ET. Taken together, these results suggest that both the cerebello-thalamo-cortical and basal ganglia-thalamo-cortical networks are involved in DT pathophysiology.

## Challenges in neuroimaging research

In an era where neuroimaging research rapidly increases, some interpretational issues should be mentioned. First, the issue of causal inference: inferring that one variable (e.g. a structural or functional change in the cerebellum) is the cause of another (e.g. tremor). Several criteria have been proposed to infer causality, such as covariation, temporal precedence, and control for “third variables”. Most neuroimaging approaches do not satisfy these criteria. For instance, cerebellar atrophy in ET patients may be related to other factors than the tremor itself (e.g. subtle cognitive or motor deficits), and it may be the consequence rather than the cause of long-standing tremor (e.g. due to reduced use of a tremulous limb). The correlation with tremor is much more specific when using concurrent EMG recordings and neuroimaging (fMRI or EEG/MEG), which increases the interpretational value of these findings. This would especially be insightful for dystonic tremor, as no study has yet focussed on tremor-related activity using EMG combined with EEG, MEG, or fMRI. A challenge that remains even with these approaches is that it is difficult to establish temporal precedence, i.e. inferring that cerebral activity precedes (or drives) the tremor. This is complicated by the limited temporal resolution of fMRI (which is in the order of seconds), and by the fact that tremor is a rhythmic process (where brain responses may be related to the previous or the next tremor cycle). There are specific approaches and advancements that address this issue. Current fMRI sequences have a limited temporal resolution which are not able to detect high-frequency neuronal oscillations at tremor frequency. Moreover, neuronal activity in specific sections of cerebral nuclei or brainstem regions cannot be accurately localized due to the limited spatial resolution of current 3T MRI scanners. Ultra-high-field MRI (at 7T) and more rapid fMRI sequences may provide the possibility to better localize tremor oscillations. Such developments have already opened the possibility to detect neuronal oscillations at 0.75 up to 2.5Hz [92,93]. Another approach to address causality is DCM, which allows one to statistically test the causal influence that one neural system exerts over the other (e.g. multiple brain regions, or muscle activity versus brain activity) [94]. DCM has already been successfully utilized in both ET and PD [17,58]. Furthermore, in EEG/MEG research, directed coherence has been used to estimate the directional flow between two coherent sources in the muscle and the brain [13]. Finally, (non-invasive) interventions combined with neuroimaging can help to infer causality. Current stimulation techniques such as TACS or TMS are often used to assess the effect of cortical neuromodulation. One shortcoming of such techniques involves the inability to affect subcortical regions effectively and selectively. Moreover, neuromodulatory effects often do not always persist after cessation of stimulation [95]. New non-invasive techniques such as transcranial focused ultrasound stimulation (TUS or FUS) have already been used to effectively manipulate cortical, subcortical, and deep cortical regions in macaques with its effects observable more than an hour after cessation of stimulation without causing permanent structural damage [95,96]. Such techniques may eventually be used in human neuroimaging research and could lead to a better understanding of the role of cortical, deep cortical or subcortical regions in the tremor network.

A second challenge in tremor research is control group selection. Some studies have compared pathological tremor to mimicked tremor in healthy controls. This is done to control for some aspects of tremor, such as the sensory consequences of the repetitive movement. However, in our view this comparison usually introduces more problems than it solves, because mimicked (voluntary) and pathological (involuntary) tremor differ in many ways. For instance, voluntary movements involve motor planning (inverse models) and altered weighing of somatosensory feedback (forward modelling), as compared to involuntary movements [97,98,99]. Furthermore, mimicked tremor phenotypically differs from pathological tremor in fundamental ways: even in subjects who were explicitly instructed to mimic essential tremor as good as possible, voluntary tremor had a lower frequency and larger wrist extension-flexion movement compared to essential tremor [16]. The comparison between different patient groups (e.g. ET versus PD) can also be problematic, since tremor occurs in different circumstances (at rest in PD versus during action in ET). Hence, resting state fMRI group-differences may be explained by the presence/absence of tremor, rather than underlying “intrinsic” differences. In these cases, use of concurrent EMG to separately model the ongoing tremor may be helpful [57,100]. In certain circumstances it may be insightful to compare two types of tremors, especially when they occur in the same patient group. For instance, many PD patients with resting tremor also exhibit a postural “re-emergent” tremor [55]. Even though rest and re-emergent tremor have similar frequencies, re-emergent tremor responds significantly less to dopaminergic medication [101]. This may be related to a larger influence of brain regions not responding to dopamine in re-emergent tremor, such as the cerebellum. A direct comparison between rest and re-emergent tremor may reveal two different tremor circuitries at play in the same patient group. Lastly, there is a lack of longitudinal studies, which would be insightful to understand disease progression and differences in degeneration or plasticity in tremor-related brain regions.

## Conclusion

Neuroimaging studies have provided convincing evidence that the cerebellum plays a key role in the pathophysiology of ET, PD tremor, and dystonic tremor syndromes. This contribution may consist of driving tremor oscillations (as an oscillator), processing tremor-related afferents, modulating activity in remote brain regions, or all of the above. In ET, the cerebellum likely acts like an oscillator, potentially due to loss of inhibitory (GABAergic) mechanisms and/or cerebellar degeneration. In addition to that, there is evidence for cerebellar dysfunction in ET, as evidenced by reduced task-related activity, reduced intrinsic (resting state) connectivity, and cerebellar atrophy. In PD, there is emerging evidence that the cerebellum is causally involved in specific tremor subtypes, i.e. in postural tremor and in dopamine-resistant rest tremor. Furthermore, the cerebellum is involved in processing tremor-related afferents in PD rest tremor, and this may be one factor that maintains the reverberation of tremor oscillations within the cerebello-thalamo-cortical circuit. In dystonia, more research is needed to define the role of the cerebellum within a larger network that also involves the basal ganglia and cortical motor regions.
